# Development of pharmaceutical prices in the Republic of Macedonia in comparison to selected countries outside the national reference baskets

**DOI:** 10.1186/2052-3211-8-S1-P12

**Published:** 2015-10-05

**Authors:** Kristina Hristova, Claudia Habl

**Affiliations:** 1 Pharmacy Sector, Health Insurance Fund (HIF), Skopje, 1000, Republic of Macedonia; 2 Development of Health Economics, Gesundheit Österreich GmbH (Austrian Public Health Institute), Vienna, 1010, Austria

## Background

Budgetary constraints - with annual growth rates that peaked at 20% - fuelled by the upcoming economic crisis demanded a change in the pricing model for pharmaceuticals in the Republic of Macedonia. Several neighbouring countries were faced with similar challenges, so the Republic of Macedonia carefully looked into their policies and thus introduced external reference pricing in 2011, which led to a reduction of prices. The specific was that both the Ministry of Health for the "unique price" (i.e. the maximum price) and the Health Insurance Fund as a basis for reimbursement performed price comparisons. The country basket for the Ministry of Health includes Bulgaria, Croatia, France, Germany, Greece, the Netherlands, Poland, Russia, Serbia, Slovenia, Turkey and the UK whereas the HIF references Bulgaria, Croatia, Serbia and Slovenia.

## Objectives

To show the trend of price reductions achieved for selected pharmaceuticals in the Republic of Macedonia as compared with the non-basket countries Hungary, Czech Republic and Austria.

Policies targeted: Analysis of the effects of external price referencing in different countries.

Stakeholders: Pricing and reimbursement authorities of the analysed countries.

Region covered: International comparison focussing on Central and Eastern Europe countries.

## Methods

Ex-post cross-country evaluation of pricing trends between 2009 and 2014 for selected out-patient medicines that are reimbursed by the Macedonian HIF using the national Macedonian price database and the European Integrated Price Database EURIPID.

## Results

The prices of the selected medicines were reduced in all analysed countries but the extents of tje decreases were different. It was also notable that some of the initially selected medicines (based on the Macedonian reimbursement list) were not marketed or reimbursed in the countries of comparison.

## Conclusions

The analysis showed that a more regular exchange of information and pricing policies could generate higher savings for national pricing and reimbursement authorities as the price development of similar products was quite diverse.

